# Theory and Simulation for Traffic Characteristics on the Highway with a Slowdown Section

**DOI:** 10.1155/2015/757823

**Published:** 2015-05-18

**Authors:** Dejie Xu, Baohua Mao, Yaping Rong, Wei Wei

**Affiliations:** ^1^MOE Key Laboratory for Urban Transportation Complex Systems Theory and Technology, Beijing Jiaotong University, Beijing 100044, China; ^2^Integrated Transport Research Center of China, Beijing Jiaotong University, Beijing 100044, China

## Abstract

We study the traffic characteristics on a single-lane highway with a slowdown section using the deterministic cellular automaton (CA) model. Based on the theoretical analysis, the relationships among local mean densities, velocities, traffic fluxes, and global densities are derived. The results show that two critical densities exist in the evolutionary process of traffic state, and they are significant demarcation points for traffic phase transition. Furthermore, the changing laws of the two critical densities with different length of limit section are also investigated. It is shown that only one critical density appears if a highway is not slowdown section; nevertheless, with the growing length of slowdown section, one critical density separates into two critical densities; if the entire highway is slowdown section, they finally merge into one. The contrastive analysis proves that the analytical results are consistent with the numerical ones.

## 1. Introduction

Traffic flow is a kind of self-driven many-particle system of strongly interacting vehicles, and it has complexly dynamic properties [1]. Traffic jams are the typical features of traffic flow; in order to study the traffic jams, several classical traffic models including car-following model, cellular automaton models, gas kinetic models, and hydrodynamic models have been proposed, and some meaningful results have been obtained. In general terms, there are two major types of traffic jams: (1) spontaneous jam which propagates backward as the stop- and go-wave; (2) stationary jam which is induced by the different kinds of bottlenecks. As for bottlenecks, the most important kinds of them are so-called flow-conserving and non-flow-conserving bottlenecks [2, 3]. When passing flow-conserving bottlenecks all vehicles pass from upstream road section to its immediate downstream road section; no vehicle leaves or enters, while non-flow-conserving bottlenecks contain sources and sinks constituted by on-ramps, off-ramps, tunnels, intersections, and so on [4–7].

On one hand, as one kind of flow-conserving bottlenecks, slowdown section reduces the local road capacity due to speed limits, which will lead to different traffic states or properties up to a certain point, such as traffic jam. Nagai et al. [8, 9] found that the spontaneous jam does not appear when the sensitivity of drivers is higher than a critical value, while the stationary jam induced by slowdown still occurs. Tanaka et al. [10] used a two-stage optimal velocity model to study the formation of jam induced by slowdown. It has been shown that the flow increases linearly with density, while it saturates at some values of intermediate density. When the flow saturates, the discontinuous front (stationary shock wave) appears before or within the slowdown section. The structure and formation of traffic jams in the two-lane highway have been studied by simulation when the bus prevents normal vehicles from moving fast in the first lane and the normal vehicles overtake the bus by changing the lane. As for non-flow-conserving bottlenecks, Lee et al. [11, 12] studied the structures of jams for on-ramp traffic flow, and the realistic dynamic models have exhibited the spatiotemporal dynamics of congested traffic states: homogeneous congested traffic, oscillatory congested traffic, triggered stop- and go-traffic, and pinned localized cluster. Jiang et al. [13] studied the on-ramp system using the cellular automata model through considering the influence of on-ramp flow on the main road and the opposite influence, whose numerical simulations show that two different types of phase diagrams exist. Kerner [14] studied the traffic states influenced by speed control strategies based on the three-phase traffic theory.

On the other hand, the dynamic slowdown sections resulting from variable speed limits are treated as one of the road-based optimization measures of traffic flow, which can increase the efficiency and stability of traffic flow when the infrastructure and the traffic demand are fixed [2, 15, 16]. Firstly, speed limits homogenize traffic flow with respect to the speed distribution; secondly, they reduce the frequency of lane changes; that is, the majority of random lane changes are no longer made since most of them are no longer associated with a significant incentive. Zhang et al. [17] found that variable speed limits can decrease traffic congestion and accident risk due to rainfall and can improve freeway traffic efficiency and safety under rainy weather. Lee et al. [18] found that variable speed limits could reduce crash potential by 5–17%, by temporarily reducing speed limits during risky traffic conditions when crash potential exceeded the prespecified threshold. This means that speed limits help prevent or delay traffic breakdowns.

For the above-mentioned research results, we learn that the study of traffic phase transition induced by slowdown section applying the analytical method is relatively less, and the theoretical relationships of the basic parameters of traffic flow and their changing rules are still not very clear. Therefore, we attempt to use the analytical method to deduce the theoretical relationships and their changing laws; furthermore, how the slowdown section influences the critical densities is also studied. This contribution is organized as follows: the new model will be presented in Section 2. Simulations and result discussions are carried out in Section 3. Finally, the conclusions are given in Section 4.

## 2. Model

**Figure 1:**
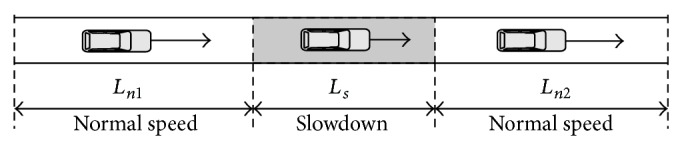
Schematic illustration of the traffic model for single-lane highway with a section of slowdown.

The CA model we are using is a modified Nagel-Schreckenberg model (one car occupies 7 cells instead of one) in the deterministic limit [19], and the speed-drop section is introduced by locally reducing the maximum speed parameter *V*_max⁡_. Without loss of generality, we consider the vehicular traffic flowing on the single-lane highway with a section of slowdown. Vehicles move with no passing on the single-lane roadway under periodic boundary condition. When the vehicles enter into a section of slowdown, we assume that the vehicles are forced to decelerate their speeds.  Figure 1 shows the schematic illustration of the traffic model for the single-lane highway with a section of slowdown that is illustrated by gray color. On the slowdown section, the velocities of vehicles must be less than the limit value, while they move with their expected speeds on normal road. The total length of the road is *L* = *L*_*s*_ + *L*_*n*_, where *L*_*s*_ is the length of the speed limit section, *L*_*n*_ = *L*_*n*1_ + *L*_*n*2_ is the length of the normal-speed sections, and *R*_*s*_ = *L*_*s*_/*L* is length ratio.

In this paper, the rules of the CA model for a parallel update are as follows.

*(i) Acceleration.* If vehicle located on the normal section:

(1) $$\begin{array}{c} V_{n}\left(\;t\;\right)=\min\left(\;V_{n}\left(\;t\;\right)+a,V_{n\max}\;\right); \end{array}$$

else

(2) $$\begin{array}{c} V_{n}\left(\;t\;\right)=\min\left(\;V_{n}\left(\;t\;\right)+a,V_{s\max}\;\right). \end{array}$$

*(ii) Deceleration. *Consider

(3) $$\begin{array}{c} V_{n}\left(\;t\;\right)=\min\left(\;V_{n}\left(\;t\;\right),{gap}_{n}\left(\;t\;\right)\;\right). \end{array}$$

*(iii) Randomization Deceleration with Probability P*. Consider

(4) $$\begin{array}{c} V_{n}\left(\;t\;\right)=\min\left(\;V_{n}\left(\;t\;\right),{gap}_{n}\left(\;t\;\right)\;\right). \end{array}$$

*(iv) Position Movement. *Consider

(5) $$\begin{array}{c} X_{n}\left(\;t\;\right)=X_{n}\left(\;t\;\right)+V_{n}\left(\;t\;\right), \end{array}$$

where *V*_*n*_(*t*) is the velocity of *n*th car at time *t*, *V*_*n*max⁡_ is the maximal velocity on the normal section, and *V*_*s*max⁡_ is the maximal velocity on the slowdown section; here *gap*_*n*_(*t*) = *X*_*n*+1_(*t*) − *X*_*n*_(*t*) − *L*_car_ denotes the distance between *n*th car and *n* + 1th car at time *t*. *L*_car_ is the length of vehicles, *a* is the vehicular acceleration, *P* denotes the randomization deceleration probability, and *X*_*n*_(*t*) indicates the position of *n*th car at time *t*.

**Figure 2:**
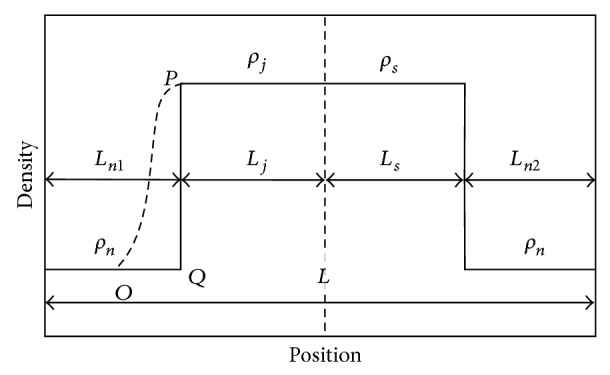
The schematic diagram of local mean densities and position.

In the following simulations, we take *V*_*n*max⁡_ = 30 cells/s, *V*_*s*max⁡_ = 10 cells/s, the length of each cell is 1 m, *L*_car_ = 7 cells, time step is *T*_step_ = 1 second, the system scale is *L* = 6000 cells, *R*_*s*_ = 0.1, *P* = 0, and *a* = 20. Here, the acceleration *a* = 20 is high compared to reality, but we still set *a* = 20 because we found that if the acceleration is lower, the local mean densities are very difficult to calculate. As will be shown in Figure 2, which is a model for the density distribution against position, the density boundary (QP) will be a curve (OP) if acceleration is lower, so the closed shape (OPQ) is not a regular triangle, whose area, that is, the number of cars, is not very easy to get, which leads to a large error. However, when the parameter *a* = 20, the curve OP becomes vertical to horizontal axis and the shape OPQ will evolve to a line QP, the boundary of normal and slowdown (or jammed) section seems more clear, and results are also more accurate. In addition, here we consider that drivers will steer their cars under an ideal circumstance, and in this paper we do not focus on the spontaneous jam but on the traffic jam induced by slowdown section. These are why the acceleration is higher than reality.

Figure 2 displays that the entire road is divided into three sections: normal section, slowdown section, and congestion section considering the existence of jam queue. The jam length is *L*_*j*_ = *R*_*j*_ *L*, here *R*_*j*_ is the ratio of the jam length to the entire road length. With the definition of the density, assuming that the starting position of slowdown section is 0 and the global density *ρ* is a constant, consequently, one can obtain the number of vehicles according to the conservation of vehicles as follows:

(6) Lρ=∫0RsLρsxdx+∫RsLRs+RjLρjxdx+∫Rs+RjLLρnxdx,

where *ρ*_*s*_(*x*), *ρ*_*j*_(*x*), and *ρ*_*n*_(*x*), respectively, denote the density functions of slowdown section, jammed section, and normal section. In this paper, we have supposed that the slowdown probability *P* = 0 in CA model, so the density functions are linear as shown in Figure 2.

## 3. Simulation and Analysis

The assumed configuration of slowdown section is displayed in Figure 1. It is obvious that the simulation consequences are not affected by the exact position of the slowdown section in the whole road, since the periodic boundary condition is adopted [7]. Initially, all vehicles are distributed on the road with uniform headway, so the global density is *ρ* = *NL*_car_/*L*, *N* is the number of vehicles. The traffic flow is defined as *F* = *ρ*〈*V*〉 with the mean velocity 〈*V*〉 = ∑*V*_*i*_/*N*, and the mean local density *ρ*_*l*_(*k*, *t*) of cell *k* at time *t* has been taken as

(7) $$\begin{array}{c} \rho_{l}\left(\;k,t\;\right)=\frac{1}{\delta }\overset{\delta -1}{\underset{i=0}{\sum }}\beta_{k+i}\left(\;t\;\right)L_{\text{car}}, \end{array}$$

where

(8) $$\begin{array}{c} \beta_{k+i}\left(\;t\;\right)=\left\{\begin{array}{cc} 1 & \text{if site }k+i\text{ is occupied at time }t\\ 0 & \text{otherwise}. \end{array}\right. \end{array}$$

The parameter *δ* denotes the length of interval on which the local density has to be computed. Similarly, we define the mean local velocity *V*_*l*_(*k*, *t*) of cell *k* at time *t* as follows:

(9) $$\begin{array}{c} V_{l}\left(\;k,t\;\right)=\frac{\sum_{i=0}^{\delta -1}\beta_{k+i}\left(t\right)V_{k+i}\left(t\right)}{\sum_{i=0}^{\delta -1}\beta_{k+i}\left(t\right)}, \end{array}$$

where *V*_*k*+*i*_(*t*) is the velocity of cell *k* + *i* at time *t*, we set *δ* = 2 × *V*_*n*max⁡_ cells. In order to avoid the transient effect, we have made a statistical averaging until the traffic flow reaches a stable state, and we average over 20 simulation runs with the above parameters unless otherwise mentioned.

### 3.1. The Relationships between Local Densities and Global Density

When the ratio of jam queue *R*_*j*_ = 0, it means that queuing phenomenon has not arisen when the density is low. Since the total number of vehicles is conserved [20], then, according to (6), the following relationship holds:

(10) $$\begin{array}{c} L\rho =R_{s}L\rho_{s}+\left(\;1-R_{s}\;\right)L\rho_{n}. \end{array}$$

Since the traffic flow of slowdown section is equivalent to that of the normal one, that is, *V*_*s*_ *ρ*_*s*_ = *V*_*n*_ *ρ*_*n*_, where *V*_*n*_ and *V*_*s*_, respectively, represent local mean velocities of the normal and slowdown section, then one gets

(11) ρnVsρRsVn−Vs+Vs,

(12) ρs=VnρRsVn−Vs+Vs.

If *V*_*n*_, *V*_*s*_, and *R*_*s*_ keep stable, we know that the local densities *ρ*_*n*_ and *ρ*_*s*_ are linear functions of the global car density *ρ*. For a special case, when *V*_*n*_ = 3*V*_*s*_, one gets *ρ*_*n*_ = *ρ*/(1 + 2*R*_*s*_), *ρ*_*s*_ = 3*ρ*_*n*_; these results are consistent with the simulation results in Figure 4.

When the ratio of jam queue *R*_*j*_ ≠ 0, it means that the queuing phenomenon occurs when the density is relatively high; one obtains

(13) $$\begin{array}{c} \rho =R_{s}\rho_{s}+R_{j}\rho_{j}+\left(\;1-R_{s}-R_{j}\;\right)\rho_{n}. \end{array}$$

According to the simulations, we know that *ρ*_*s*_ = *ρ*_*j*_ when the jammed queue appears. So the integrating of the jam queue and the original slowdown section can be regarded as a new slowdown section, whose length grows with increasing density. Similarly, one obtains

(14) ρnρRs+RjVn/Vs−1+1,

(15) ρs=ρRs+Rj1−Vs/Vn+Vs/Vn

or

(16) $$\begin{array}{c} R_{s}+R_{j}=\left(\;\frac{V_{s}}{{V_{n}-V}_{s}}\;\right)\left(\;\frac{\rho -\rho_{n}}{\rho_{n}}\;\right). \end{array}$$

When substituting *R*_*j*_ = 0 into (14), (11) is equal to (15). In the same way, for a specific situation, when *V*_*n*_ = 3*V*_*s*_, one gets *ρ*_*n*_ = *ρ*/[1 + 2(*R*_*s*_ + *R*_*j*_)], *R*_*s*_ + *R*_*j*_ = (*ρ* − *ρ*_*n*_)/(2*ρ*_*n*_).

In addition, one may pay more attention to the critical densities of the traffic phase transition. When the global density reaches the first transition point, the queue starts to appear; when it comes to the second one, the queue extends to the full road. Therefore, we separately define the two transition points as the first critical density *ρ*_*c*1_ and the second critical density *ρ*_*c*2_. According to the definition, the global density *ρ* = *ρ*_*c*1_ when *R*_*j*_ = 0, and *ρ* = *ρ*_*c*2_ when *R*_*s*_ + *R*_*j*_ = 1. On the basis of these analyses, we can derive the following:

(17) ρc1RsVnVs−1+1ρn,ρc2=VnVsρn.

After achieving the expressions of critical densities, a comprehensive analysis about the changing rules of local mean densities can be given.

(a) If 0 < *ρ* ≤ *ρ*_*c*1_, the local densities *ρ*_*n*_ and *ρ*_*s*_ are linear functions of the global density *ρ*, and when *ρ* = *ρ*_*c*1_, one gets

(18) ρnρc1Vsρc1RsVn−Vs+Vs,ρsρc1=Vnρc1RsVn−Vs+Vs.

(b) If *ρ*_*c*1_ < *ρ* ≤ *ρ*_*c*2_, the traffic jam begins to form, and the upper road ahead of the limit section is congested traffic flow, while the downstream road is free flow. In such a case, the density of the free flow *ρ*_*n*_ and that of the limit section *ρ*_*s*_ are staying the same with growing density.

(c) If *ρ*_*c*2_ < *ρ* ≤ 1, the traffic of the whole road is congested state and *R*_*s*_ + *R*_*j*_ = 1, *V*_*n*_ = *V*_*s*_, and *ρ*_*n*_ = *ρ*_*s*_. Substituting them into (14), the following formula can be derived:

(19) $$\begin{array}{c} \rho_{n}=\rho_{s}=\rho . \end{array}$$

**Figure 3:**
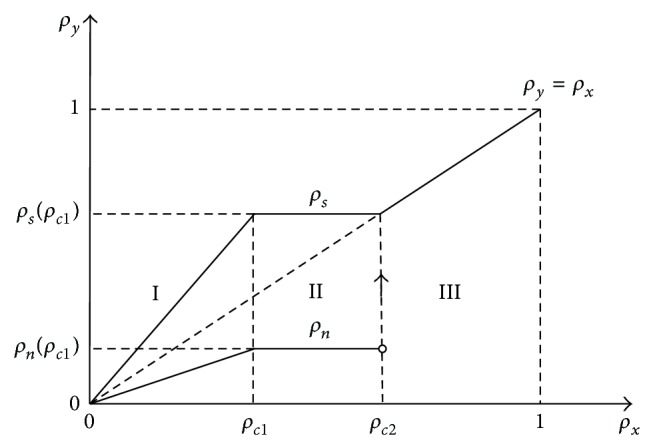
The theoretical relationships of local densities against global density.

The theoretical relationships between the local densities and the global density are shown in Figure 3. As one can see, it is divided into three regions. In region I, the local densities *ρ*_*n*_ and *ρ*_*s*_ are linear functions of the global car density *ρ*; in region II, *ρ*_*n*_ and *ρ*_*s*_ still have the same value; at the transition point of regions II and III, the density *ρ*_*n*_ has an instantaneous jump (the open circle denotes when the density *ρ* = *ρ*_*c*2_, *ρ*_*n*_ leaps to *ρ*_*s*_); this is because when the global density *ρ* = *ρ*_*c*2_, the free flow disappears and the entire road becomes congested; in region III, two local densities *ρ*_*n*_ and *ρ*_*s*_ are equal to the global density *ρ*. In a word, when the density is less than the first transition point, all vehicles of their own road section move freely with the same headway and the slowdown section does not affect the vehicles on the regular road sections. When the density is higher than the first transition point and less than the second transition point, the traffic jam occurs before the slowdown section. When the density is higher than the second transition point, all vehicles move slowly, and the traffic state results in the homogeneous congested traffic; therefore the local densities are equal to the global density.

**Figure 4:**
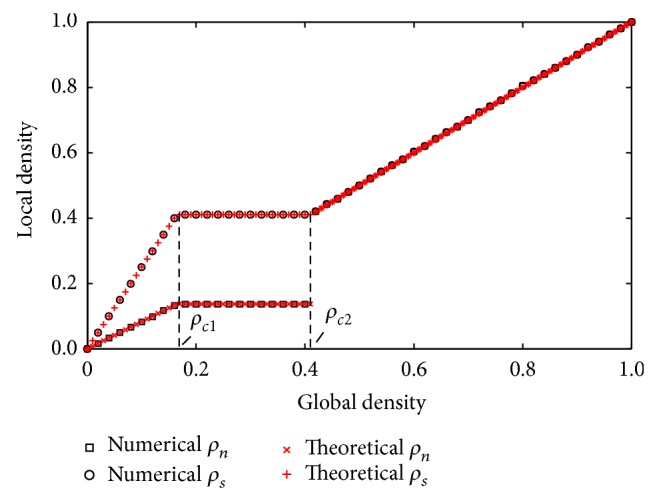
The simulation relationships of local densities against global density.

Figure 4 shows the plot of local densities against global density. The black open squares and open circles represent the simulation results, and the red crosses represent the theoretical results, where *ρ*_*n*_ = 0.137, *ρ*_*c*1_ = 0.164, and *ρ*_*c*2_ = 0.411. The numerical simulations results agree with those obtained by the analytical method.

### 3.2. The Relationships between the Traffic Flux, Local Velocities, and Global Density

  (a) If 0 < *ρ* ≤ *ρ*_*c*1_, the flow *F* can be expressed as follows:
  (20) $$\begin{array}{c} F=\rho_{n}V_{n}=\frac{V_{n}V_{s}\rho }{\left[R_{s}\left(V_{n}-V_{s}\right)+V_{s}\right]} \end{array}$$
 or
  (21) $$\begin{array}{c} F=\rho_{s}V_{s}=\frac{{V_{s}V}_{n}\rho }{\left[R_{s}\left(V_{n}-V_{s}\right)+V_{s}\right]}. \end{array}$$

When the global density is low, the number of vehicles is relatively less, and the mutual influences among vehicles are very weak and they can move freely; thus the local velocities *V*_*n*_ and *V*_*s*_ are independent of global density; that is, *V*_*n*_ = *V*_*n*max⁡_, *V*_*s*_ = *V*_*s*max⁡_, while the local densities *ρ*_*n*_ and *ρ*_*s*_ linearly grow with increasing density *ρ*, so the traffic flow grows linearly against global density *ρ* as well.

 (b) If *ρ*_*c*1_ < *ρ* ≤ *ρ*_*c*2_, the flux can be calculated by
  (22) $$\begin{array}{c} F=\rho_{n}V_{n}=V_{s}\rho_{s}. \end{array}$$

Under this circumstance, the jam has formed, and the flux reaches saturation, but the vehicles can still move at highest velocity *V*_*n*max⁡_ except for the speed restriction and jammed sections; thus the local velocities *V*_*n*_ and *V*_*s*_ are also independent of global density. According to the preceding analyses, the density has a steady value; therefore the flux is also independent of global density and keeps constant.

 (c) If *ρ*_*c*2_ < *ρ* ≤ 1, the traffic of entire road is congested state. Firstly, we are sure that if the global density *ρ* = *ρ*_*c*2_ and *ρ* = 1, then flux *F* = *ρ*_*c*2_ *V*_*n*_ and *F* = 0, respectively. Additionally, we find out that the traffic flux is linearly decreasing against global density, so we can deduce the formula of flux:
  (23) $$\begin{array}{c} F=-\frac{\rho_{n}V_{n}}{1-\rho_{c2}}\left(\;\rho -\rho_{c2}\;\right)+\rho_{n}V_{n}. \end{array}$$

When the density *ρ* satisfies *ρ*_*c*2_ < *ρ* ≤ 1, we have known that *ρ*_*n*_ = *ρ*_*s*_ = *ρ*; then by solving (23), one obtains

(24) $$\begin{array}{c} V_{n}=V_{s}=\frac{1}{\rho }\left(\;\rho_{n}V_{n}+\frac{\rho_{n}V_{n}\rho_{c2}}{1-\rho_{c2}}\;\right)-\frac{\rho_{n}V_{n}}{1-\rho_{c2}}. \end{array}$$

**Figure 5:**
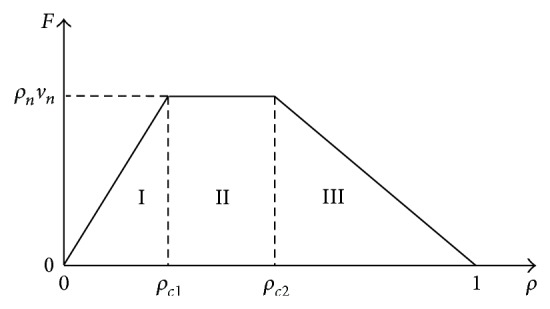
Theoretical curve of density-flux.

**Figure 6:**
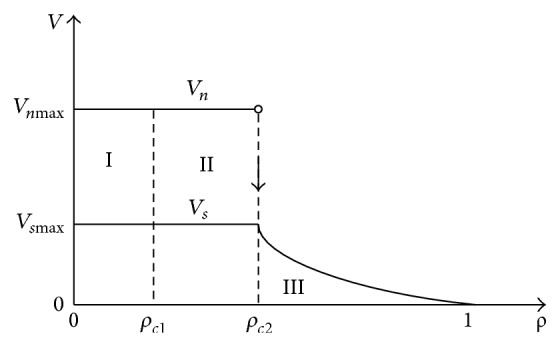
Theoretical curves of local velocities against global density.

In summary, we get the theoretical density-flow relation and velocity-density relation, as shown in Figures 5 and 6. One can see that the two plots are separated into three regions. In region I, all vehicles move with their desired velocities; the traffic flow is in the free traffic; therefore the flux increases linearly with density; in region II, vehicles are jammed before the slowdown section, and the flux is limited to the capacity of slowdown section, so it maintains in a definite value *F* = *ρ*_*n*_ *V*_*n*_; in region III, vehicles move with the same value of short headway, and they are distributed uniformly on the ring road. Thus, the flux decreases linearly. Unlike the basic diagram, the velocities *V*_*n*_ and *V*_*s*_ keep constant in regions I and II; and there is a jump of velocity *V*_*n*_ like Figure 3. In particular, they are inversely proportional to global density.

**Figure 7:**
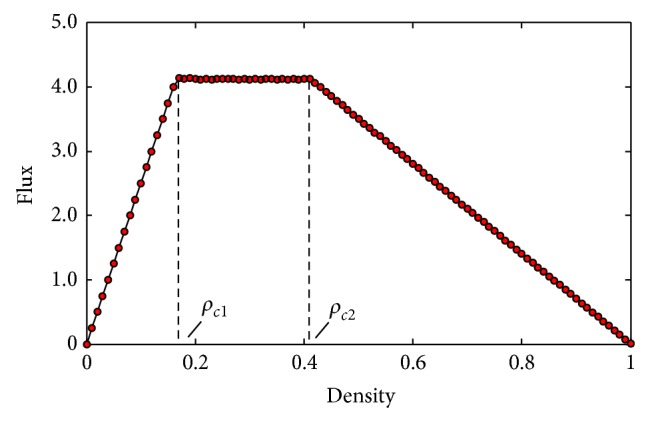
The numerical simulations curve of density-flux.

Figure 7 shows the simulation density-flow result. We get that the first critical density *ρ*_*c*1_ = 0.17 and the second critical density *ρ*_*c*2_ = 0.41; yet the theoretical critical densities *ρ*_*c*1_ = 0.164, *ρ*_*c*2_ = 0.411, which are very close to the simulation results. However, there is a tiny deviation, which is caused by the density step because we set it as 0.01 in numerical simulations. When the global density *ρ* = 0.16, the traffic state is free flow; when it increases to 0.17, the jam turns out, but the global density is not the density that jam just begins to raise, so the simulation result is slightly greater than the theoretical one. Inversely, when the global density *ρ* = 0.41, the traffic is not completely jammed; while it grows to 0.42, the traffic has been entirely congested; thus the simulation critical density is less than the theoretical one. The theoretic saturated flow is *F*_max⁡_ = *ρ*_*n*_ *V*_*n*_ = 0.137 × 30 = 4.11, which is in accordance with the numerical simulations results.

**Figure 8:**
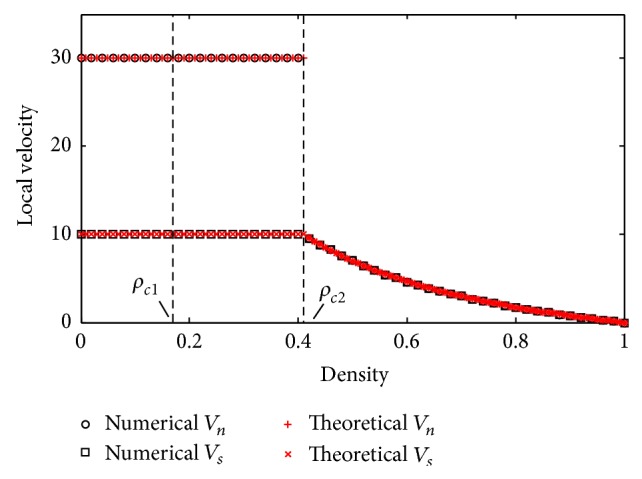
Numerical simulations curve of local velocities against global density.

Figure 8 shows the density-velocity relation through numerical simulations. The black open squares and open circles, respectively, indicate the mean local speed of limit section and that of normal section except the jam queue; the red crosses denote their theoretical consequences. From the diagram, when the global density *ρ* < *ρ*_*c*2_, the speeds keep steady; when *ρ* ≥ *ρ*_*c*2_, they gradually decrease with *ρ*.

### 3.3. The Total Ratio of the Jammed Queue and the Section with Speed Limitation

In (16), we order a parameter *R* = *R*_*s*_ + *R*_*j*_, and *R* is the total ratio which is given by the summary length of queue and slowdown section divides that of the whole road. Here the changing law of the ratio is analyzed under two conditions.

*(a) R*_*s*_ > 0. When the global density *ρ* ≤ *ρ*_*c*1_, the traffic is free flow, so the ratio *R*_*j*_ = 0 and the total proportion *R* = *R*_*s*_; when the global density *ρ*_*c*1_ < *ρ* ≤ *ρ*_*c*2_, the queue ration *R*_*j*_ has linear relation with the global density growing, and when *ρ* = *ρ*_*c*2_, *R*_*j*_ reaches its peak value 1 − *R*_*s*_; that is, *R* = 1. 

*(b) R*_*s*_ = 0. In such situation, it means that the whole road is homogeneous road section and the two critical densities emerge into one which is named *ρ*_*c*_; when *ρ* ≤ *ρ*_*c*_, the ratio *R* = 0; once the density *ρ* > *ρ*_*c*_, the ratio *R* = 1.

**Figure 9:**
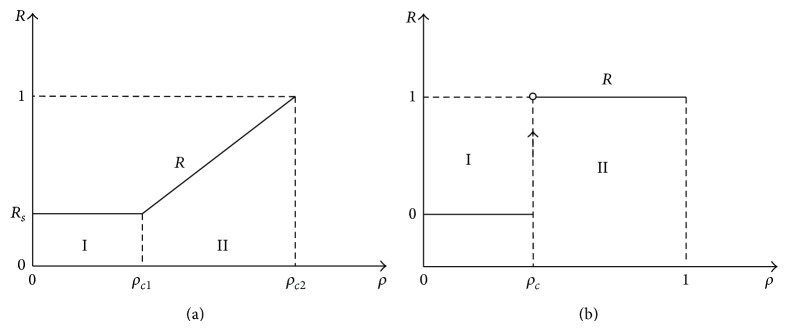
The total ratio of jam and slowdown section. (a) *R*_*s*_ > 0, (b)  *R*_*s*_ = 0.

Figure 9 shows the plots of curve *R*, Figure 9(a) corresponds to the case when *R*_*s*_ > 0, and Figure 9(b) denotes the case when *R*_*s*_ = 0. One can see that the curve *R* is divided into two regions. In Figure 9(b), when the global density *ρ* = *ρ*_*c*1_, the total ratio *R* is zero, and the open circle indicates that the ratio *R* is 0 rather than 1.

**Figure 10:**
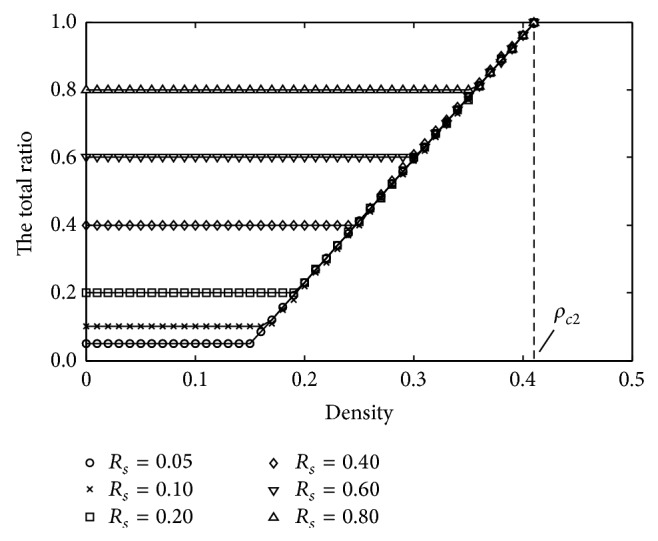
Plots of total ratio against the density for various proportions of slowdown section.

Figure 10 shows the simulation consequences of total ratio *R*. The solid lines represent the theoretic data with different *R*_*s*_, respectively, and others indicate simulation results. One can know that the first critical density continuously increases with the growing of ratio *R*_*s*_, and it has a trend of approaching the second critical density which is always constant. When the critical density *ρ* = *ρ*_*c*2_, the ratio *R* reaches maximum 1, which conforms to the theoretical inferences. Based on the trend, the first critical density comes to the second one, so we further probe the relationships between *R*_*s*_ and two critical densities through the basic diagram.

**Figure 11:**
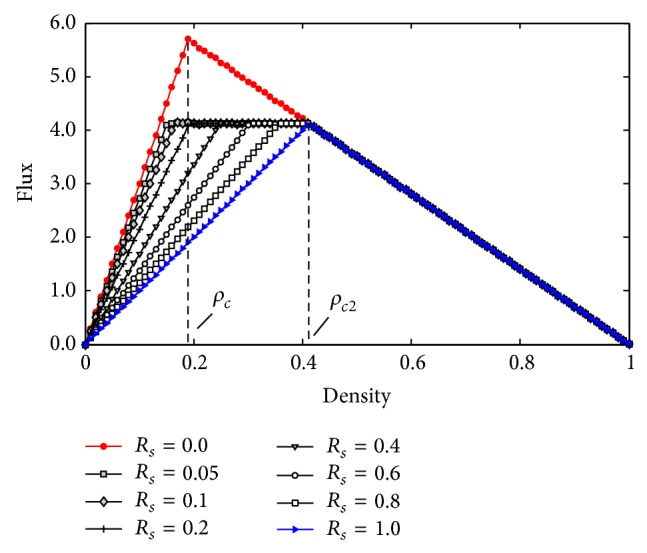
Plots of flux against density for different ratio of slowdown section.

Figure 11 is the plot of flux against density with different ratio *R*_*s*_. It shows that when *R*_*s*_ = 0 (the red curve) or *R*_*s*_ = 1 (the blue curve), there is only one critical density, which can be calculated by the formula *ρ*_*c*_ = *L*_car_/(*L*_car_ + *V*_max⁡_); here *V*_max⁡_ is the maximal speed. When *R*_*s*_ = 0, that is, there is no slowdown section on the highway and the critical density *ρ*_*c*_ = *L*_car_/(*L*_car_ + *V*_*s*max⁡_) = 0.189 ≈ 0.19, the saturated flow is *F*_max⁡_ = *ρ*_*c*_ *V*_*n*max⁡_ = 5.7; when *R*_*s*_ = 1, the entire road is slowdown section, so the critical density *ρ*_*c*_ = *L*_car_/(*L*_car_ + *V*_*n*max⁡_) = 0.411, and the saturated flow is *F*_max⁡_ = *ρ*_*c*_ *V*_*s*max⁡_ = 4.1, while when the ratio *R*_*s*_ ∈ (0, 1), there are two critical densities. These results suggest that the capacity of the regular road section determines its maximum flux when the road section is homogeneous, while if road exists in inhomogeneous sections (e.g., slowdown sections or other bottlenecks), the capacity is restricted to the bottlenecks. In addition, it should be noted that these results derive from a closed single-lane ring road populated with identical drivers and vehicles, and the global density is the only control parameter. In contrast, the real road networks are open. Nevertheless, this investigation will be beneficial for us to draw far-reaching conclusions on more realistic open systems.

## 4. Conclusions

Through applying the analytical method and the numerical simulation, we deduced out the theoretical relationships of local mean densities, local mean velocities, and flux against global density *ρ*. In addition, we found that two critical densities appear in the theoretical curves when a slowdown section exists on a highway, and they can be divided into three regimes by the critical densities.

- 0 < *ρ* ≤ *ρ*_*c*1_: the traffic is free flow. The local densities and flux grow linearly; the local velocities remain steady.
- *ρ*_*c*1_ < *ρ* ≤ *ρ*_*c*2_: the congestion has formed, and the flux comes to saturation. The local densities, local velocities, and saturation flux all maintain a stable value, and the local densities and velocities have definitive mathematic relation depending on the critical density *ρ*_*c*1_.
- *ρ*_*c*2_ < *ρ* ≤ 1: the traffic of entire road is congested. The local densities continue to grow and they are equal to the global density *ρ*; the flux decreases linearly; and the two local densities are inversely proportional to global density *ρ*.

Additionally, the changing laws of two critical densities with different length of slowdown section suggest that there is only one critical density when the ratio *R*_*s*_ = 1; when the ratio *R*_*s*_ ∈ (0,1), there are two critical densities and the first one increases gradually with the growing of *R*_*s*_, but the second one keeps stable.

In the present paper we have devoted ourselves to studying the situation under deterministic rule. To study the cases where the randomization is considered will be future work.
